# Temperature and particulate matter as environmental factors associated with seasonality of influenza incidence – an approach using Earth observation-based modeling in a health insurance cohort study from Baden-Württemberg (Germany)

**DOI:** 10.1186/s12940-022-00927-y

**Published:** 2022-12-16

**Authors:** Jörn Rittweger, Lorenza Gilardi, Maxana Baltruweit, Simon Dally, Thilo Erbertseder, Uwe Mittag, Muhammad Naeem, Matthias Schmid, Marie-Therese Schmitz, Sabine Wüst, Stefan Dech, Jens Jordan, Tobias Antoni, Michael Bittner

**Affiliations:** 1 Institute of Aerospace Medicine, German Aerospace Center (DLR), 51147 Cologne, Germany; 2 Department of Pediatrics and Adolescent Medicine, University Hospital Cologne, Cologne, Germany; 3 German Remote Sensing Data Center, German Aerospace Center (DLR), Oberpfaffenhofen, Germany; 4 Allgemeine Ortskrankenkasse Baden-Württemberg (AOK-BW), Stuttgart, Germany; 5 Kohat University of Science and Technology, Kohat, Pakistan; 6 Institute of Medical Biometry, Informatics and Epidemiology, University Hospital Bonn, Bonn, Germany; 7 Medical Faculty, University of Cologne, Cologne, Germany

**Keywords:** Air quality, Epidemiology, Disease burden, Infectious disease

## Abstract

**Background:**

Influenza seasonality has been frequently studied, but its mechanisms are not clear. Urban in-situ studies have linked influenza to meteorological or pollutant stressors. Few studies have investigated rural and less polluted areas in temperate climate zones.

**Objectives:**

We examined influences of medium-term residential exposure to fine particulate matter (PM_2.5_), NO_2_, SO_2_, air temperature and precipitation on influenza incidence.

**Methods:**

To obtain complete spatial coverage of Baden-Württemberg, we modeled environmental exposure from data of the Copernicus Atmosphere Monitoring Service and of the Copernicus Climate Change Service. We computed spatiotemporal aggregates to reflect quarterly mean values at post-code level. Moreover, we prepared health insurance data to yield influenza incidence between January 2010 and December 2018. We used generalized additive models, with Gaussian Markov random field smoothers for spatial input, whilst using or not using quarter as temporal input.

**Results:**

In the 3.85 million cohort, 513,404 influenza cases occurred over the 9-year period, with 53.6% occurring in quarter 1 (January to March), and 10.2%, 9.4% and 26.8% in quarters 2, 3 and 4, respectively. Statistical modeling yielded highly significant effects of air temperature, precipitation, PM_2.5_ and NO_2_. Computation of stressor-specific gains revealed up to 3499 infections per 100,000 AOK clients per year that are attributable to lowering ambient mean air temperature from 18.71 °C to 2.01 °C. Stressor specific gains were also substantial for fine particulate matter, yielding up to 502 attributable infections per 100,000 clients per year for an increase from 7.49 μg/m^3^ to 15.98 μg/m^3^.

**Conclusions:**

Whilst strong statistical association of temperature with other stressors makes it difficult to distinguish between direct and mediated temperature effects, results confirm genuine effects by fine particulate matter on influenza infections for both rural and urban areas in a temperate climate. Future studies should attempt to further establish the mediating mechanisms to inform public health policies.

## Background

Influenza, which is caused by the influenza-A or the influenza-B virus, is transmitted through the respiratory organs via physical contact with an infectious person or with contaminated objects, or via airborne droplets or droplet nuclei, with the latter having a radius of < 2.5 µm. Up to 1 billion infections occur per year world-wide, with up to 5 million severe cases and 50,000 deaths attributable to influenza [1]. However, substantial seasonal variation exists, with infection rates culminating in winter, at least in temperate climates in the southern and northern hemisphere. Thus, influenza bears many similarities with SARS-CoV2, which has been causing the Covid-19 pandemic since December 2019.

The astounding seasonal variation appears to be related to several factors that can be grouped into four categories: pathogen abundance; environmental factors; host behavior; and host susceptibility [2]. The environmental factors, temperature, humidity, and vapor pressure have been implicated [3]. Notably, seasonality increases with latitude [4]. Thus, incidence rates depict a single peak in the temperate climate zones, with peaks in January to March in the northern hemisphere and in July to September in the southern hemisphere. Tropical climates may depict one, two (example: Hong Kong [5]), or no peak at all. The observation suggests that seasonal variation is not due to one factor alone, but rather to a combination of factors.

In addition to the meteorological factors, air pollutants such as ozone (O_3_) and sulfur dioxide (SO_2_) may modulate influenza transmission [6]. Associations of O_3_ and coarse particulate matter (PM_10_) with influenza-hospitalization, but not with influenza-mortality have been reported for Hong Kong [7] The finding has been confirmed by a pediatric cohort study in Brisbane (Australia), which also reports significant interaction between pollutant and temperature effects [8]. For Nanjing, a more temperate climate, associations were also found for nitrogen dioxide (NO_2_). A recent study from Wuhan attributes an additional role to SO_2_ [9]. Notably, that study also demonstrates the strongest linkage with a one- or two-day lag time only, suggesting a rather acute mediation of the effect. Another recent study of mortality in Milan during the winter 2016/2017 suggests that air pollution, low temperature and influenza infection jointly mediate excess mortality [10]. Moreover, re-assessment of historical data suggests that smog powerfully contributed to the death toll of London’s 1952 influenza epidemic [11]. Another study posits particulate matter (PM) arising from coal combustions severely aggravated 1918’s world-wide influenza pandemic [12]. Overall several studies have indeed examined the relationship between temperature, humidity and influenza [3, 13, 14], but only very few that propose effects of fine particulate matter (PM_2.5_) [15, 16]. However, all these studies are carried out under moderate to high air pollution conditions [7, 17] or for selected cities [15, 17, 18].

While environmental factors appear to strongly interact with each other in their impact on influenza incidence and severity, the fact that air pollutants themselves exhibit seasonality in temperate climates complicates the interpretation [19, 20]. Whether the association reflects non-causal co-variation or true causality cannot be discerned. A detailed understanding of the causative inter-relationships is particularly lacking for temperate climates, where the existing literature is based on *in-situ* ground measurements from larger cities. Furthermore, there is a general lack of studies including data that range from urban and highly-polluted to rural and less-polluted areas, thereby taking advantage of spatial variation in environmental factors in addition to their temporal variation. Spatial variation may be less error-prone, as it minimizes the effects of non-causative covariation on the observed outcome. *In-situ* ground measurements would be cumbersome to perform over wider geographical territories, but Earth observation in combination with twenty-first century computing power does allow for modeling of environmental exposure over wide-spread areas.

The German Aerospace Center as a research center (DLR) and the AOK Baden-Württemberg as the 5^th^ largest statutory health insurance formed a unique partnership in 2020 to understand the complex system of health, environment and social structures. Man-made climate change is changing decisive factors that affect the health of the population in a variety of ways. For the AOK Baden-Württemberg, it is of crucial importance to understand the effects of climate change on health in detail to make significant progress in clarifying the dose–response relationship. Particular attention is paid to the protection of vulnerable subpopulations.

We, therefore, applied a novel approach combining state-of-the-art Earth observation-based numerical modeling with health insurance data to assess effects of residential exposure to pollutants and meteorological factors on the seasonality of influenza infections in, both, rural and urban environments of a temperate climate zone. We hypothesized that concentrations of PM, NO_2_, SO_2_ and ozone would be positively associated with influenza incidence, and that temperature would be negatively associated.

## Methods

### Study population

The study was designed as an observational cohort study, making use of all residents of Baden-Württemberg insured with Allgemeine Ortskrankenkasse (AOK) in Baden-Württemberg. In Germany, each federal state has its own AOK, which is the public corporation commissioned to provide health insurance to people. Until 1996, health insurance corporations had been linked to professions in Germany, and AOK therefore has been and continues to be the main health insurer for the working class.

Baden-Württemberg is Germany’s third most populated state, with 11 million inhabitants of which 4.5 million are currently health-insured with AOK Baden-Württemberg. The state’s capital is Stuttgart, with approximately 5 million residents in its metropolitan region. Baden-Württemberg’s total area amounts approximately 36,000 km^2^ comprising highly rural and urban environments.

We included data from all AOK clients residing in Baden-Württemberg collected for the period between January 2010 and December 2018.

The study is in accordance with the declaration of Helsinki and was approved by the Ethical Committee of the Medical Council (Landesärztekammer Nordrhein) within the VARIAQ study (lfd Nr 2,020,092). Data management plans were approved by data protection officers on both sides (DLR and AOK) before data exchange commenced.

### Identification of cases

We gathered and merged at person-level health data from 5 AOK databases, as separate data bases exist for in-patient hospital cases, for out-patient hospital cases, for sick-leave, and two separate data-bases that cover data from other out-patient cases. Duplication of individual records was prevented through AOK-internal identifiers. The data sources primarily serve to manage remuneration of health care providers. As remuneration is occurring on a quarterly basis, the resulting data base contains quarterly information on diagnoses.

We classified all cases with ICD-10 codes J09 (influenza due to certain identified influenza viruses), J10 (influenza due to other identified influenza virus) or J11 (influenza due to unidentified virus with other respiratory manifestations) in a given quarter as prevalent cases. They were furthermore classified as new cases when the ICD-10 codes J09, J10 or J11 were present in a given quarter but not in the preceding quarter. From these data, we aggregated the number of new cases and the number of existing cases per quarter, per 5-digit postcode, and per gender. Sex and age information was abandoned to preclude exposure of personally identifiable information, and to be in keeping with data protection regulations. This approach was necessary in particular for remote areas with low population densities.

### Environmental data

As stated above, our goal was to model environmental exposure for the entire region of Baden- Württemberg for all quarters between 2010 and 2018. Therefore, we retrieved air pollution data, *i.e.* surface level concentrations of PM_10_, PM_2.5_, NO_2_, SO_2_ and O_3_, from the Copernicus Atmosphere Monitoring Service (CAMS) European air quality reanalyses, which is a dataset resulting from an ensemble of seven (nine, after the upgrade in October 2019) chemical transport models composed of daily forecast and analysis data on the main pollutants’ concentration. The dataset has a native temporal resolution of one hour and a horizontal resolution of 0.1° × 0.1° [21, 22]. Forecast and analysis data are annually validated and adjusted by assimilation of data from the Copernicus in-situ component like measurements from the European Environmental Agency’s (EEA) station network. The re-analysis is publicly available on the Copernicus Atmosphere Data Store (ADS, https://ads.atmosphere.copernicus.eu).

We retrieved meteorological data from the Climate Data Store (CDS) of ECMWF, a free and open access platform gathering quality assured climate data derived from Earth observation, global and regional climate re-analyses of past observations, seasonal forecasts and climate projections. Moreover, we obtained data of downward ultraviolet (UV) radiation at the surface with hourly temporal resolution and with horizontal resolution of 0.25° X 0.25° from the ERA5 reanalysis dataset. This parameter is defined as the amount of UV radiation with a wavelength of 0.2–0.44 µm reaching the surface [23]. The ERA5 dataset is produced within the Copernicus Climate Change Service (C3S) that provides records of global atmosphere, land surface and ocean from 1959 onwards [24]. We retrieved air temperature data, dewpoint temperature at 2 m above the surface, from the ERA-5-Land dataset, which is also a reanalysis dataset available on the ECMWF CDS with enhanced resolution compared to ERA5. The dataset is available for the time period from 1950 until 2–3 months before the present time. It consists of a replay of the land component of ERA5 climate reanalysis with an applied sea mask and with the assimilation of observational data [25]. This dataset offers a native temporal resolution of one hour and a horizontal resolution of 0.1°X0.1°. A summary of the data features is provided in Table 1.

**Table 1:** Summary of information on sources used to gather the environmental data used for this study

Variable	Source Dataset	Horizontal Resolution	Temporal Resolution	Unit	URL
PM_2.5_, PM_10_, O_3_, NO_2_	CAMS European air quality reanalyses	1 h	0.1°X0.1°	µg /m^3^	https://ads.atmosphere.copernicus.eu/cdsapp#!/dataset/cams-europe-air-quality-reanalyses
Downward UV radiation at the surface	ERA5 hourly data on single levels from 1979 to present	1 h	0.25° × 0.25°	J/m^2^	https://cds.climate.copernicus.eu/cdsapp#!/dataset/reanalysis-era5-single-levels
2 m temperature	ERA5-Land hourly data from 1950 to present	1 h	0.1°X0.1°	K	https://cds.climate.copernicus.eu/cdsapp#!/dataset/reanalysis-era5-land
2 m dewpoint temperature	ERA5-Land hourly data from 1950 to present	1 h	0.1°X0.1°	K	https://cds.climate.copernicus.eu/cdsapp#!/dataset/reanalysis-era5-land

From the 2 m temperature data we calculated daily aggregates of minimum, mean, and maximum. Similarly, we also converted dewpoint temperature data to °C and derived vapor pressure using an empirical formula [26], see Eq. 1. Afterwards, we produced daily minimum, mean, and maximum surface vapor pressure aggregates.

1 $$e=6.112*\mathrm{exp}( \frac{17.67*{T}_{d}}{{T}_{d}-243.5})$$

where: e = Vapor pressure in hPa, Td: dew point temperature in °C

We aggregated UV radiation data on a daily basis, calculating the sum of the radiation reaching the surface. We also converted the unit from J/m^2^ to W/ m^2^ dividing by the integration time in seconds.

For all the layers of environmental variables, we increased the horizontal resolution via a grid-granularization of the grid while using the nearest neighbor interpolation method. The new grid resolution is 0.067° X 0.067°.

To obtain geographical data, we derived shapefiles for all 5-digit postcodes in Baden-Württemberg from the ESRI Deutschland databank [27]. Geographical and demographical information in the dataset were extracted from ©OpenStreetMap contributors and from data of the 2011 census of Germany’s federal authority of statistics (https://www.zensus2011.de/DE/Home/home_node.html). The number of inhabitants in the shapefile was calculated for each polygon based on population density per 1km^2^ according to the DESTATIS’s Zensus2011 dataset. We used the shapefiles to mask environmental layers and to perform a spatial aggregation for each polygon. Thus, we obtained daily aggregates of all the environmental variables considered for each 5-digit postcode area. Finally, we performed another aggregation step to yield quarterly (i.e. three-month-averaged) data, calculating the mean values within each postcode-quarter aggregate window.

### Data scrutiny

At the stage of data exchange, records with unknown age were excluded, leading to a loss of 0.56% of observations. Such spurious records in insurance data bases typically emerge from accidents and emergency for which the identity of the patient is not fully known. Next, a check for personally identifiable information was performed, which meant that all information with less than 3 persons (including cases and non-cases) per aggregate window (postcode, quarter) had to be discarded. However, the loss amounted to 0.0032% of individual-level data only.

### Statistical methods

**Fig. 1:**
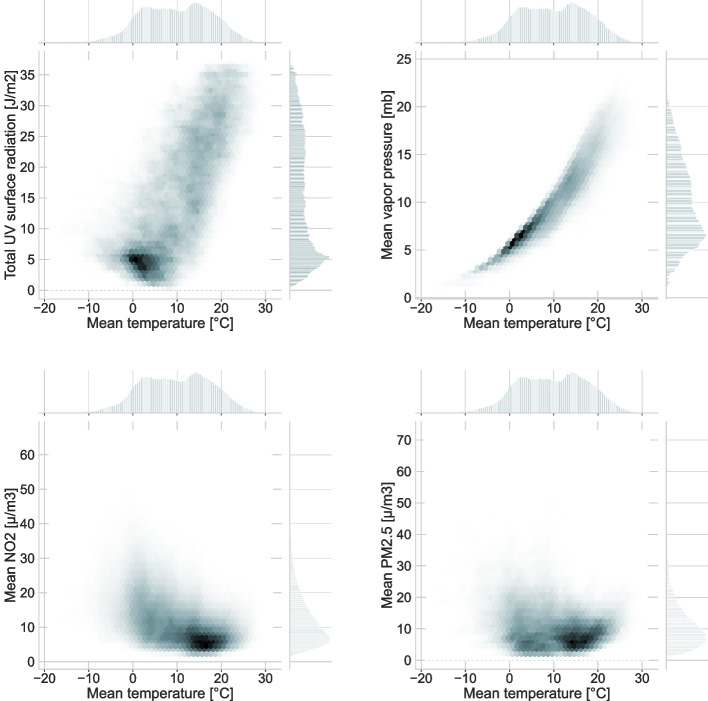
Relationship of UV radiation, NO_2_ and PM_2.5_ with temperature. Joint distribution plots for UV, vapor pressure, NO_2_ and PM_2.5_ versus temperature. The distributions shown in each plot refer to mean daily aggregates for each ZIP-code area in Baden-Württemberg between 2010 and 2018. The dataset used to produce the plots are those reported in Table 1

Before statistical model fitting, we explored pairwise collinearities among the environmental variables. According to the correlation matrix given in Table 2, we observed substantial collinearity in particular for the variables O3, PM10, UV radiation and vapor pressure (right and lower quadrants of the Table 2). Accordingly, to reduce issues arising from collinearity, we restricted statistical modeling to PM_2.5_, NO_2_, Temperature and Precipitation. Figure 1 presents joint distribution plots for environmental variables presenting collinearity.

**Table 2:** Matrix with Pearson’s correlation coefficients for environmental variables

	**PM**_**2.5**_	**NO**_**2**_	**Tempe-rature**	**Precipi-tation**	**O**_**3**_	**PM**_**10**_	**UV-Radiation**	**Vapor Pressure**
PM_2.5_		0.61	-0.59	-0.45	-0.42	**0.86**	-0.47	-0.61
NO_2_	0.61		-0.59	-0.32	**-0.78**	0.64	-0.7	-0.58
Temperature	-0.59	-0.59		0.05	**0.73**	-0.56	**0.86**	**0.98**
Precipitation	-0.45	-0.32	0.05		0.08	-0.41	0.04	0.14
O_3_	-0.42	**-0.78**	**0.73**	0.08		-0.44	**0.93**	0.66
PM10	**0.86**	0.64	-0.56	-0.41	-0.44		-0.43	-0.57
UV Radiation	-0.47	-0.7	**0.86**	0.04	**0.93**	-0.43		**0.79**
Vapor Pressure	-0.61	-0.58	**0.98**	0.14	0.66	-0.57	**0.79**	

Correlation analyses were performed with entering one value per postcode-quarter aggregate window. Values for which the squared correlation coefficient was > 0.5 have been highlighted in red, and the set of variables used for statistical modeling is shaded

With these four selected environmental independent variables, we fitted generalized additive models [28]. Generalized additive models allow for a flexible specification of outcome dependence on the variables as the predictor comprises a sum of (possibly nonlinear) smooth functions. The number of new influenza cases per post code represents count data (= non-negative integers) and hence the modeling approach was based on a negative binomial model [29] using the number of new cases as outcome variable. Regarding the independent variables, we used smooth P-spline functions for environmental variables, a Gaussian Markov random field smoother over postcodes and an offset for the number of AOK clients per postcode. Of note, entering postcode as an independent variable adjusts for site-specific traits such as population density, urbanity and other factors. In addition, we included two variables (‘quarter’ and ‘season’) to model temporal effects. Firstly, we used quarter as a means of accommodating seasonal variation that is not necessarily independent of environmental stressors under study. However, as much as stressors vary with ‘quarter’, this approach may not fully capture the true stressor effects. Therefore, we built a full model that does include quarter (called *full* model henceforth), and another model that does not include quarter (henceforth called *restricted* model). Accordingly, the restricted model attempts to explain seasonal variation in influenza incidence by seasonal variation in environmental stressors only. As a second temporal variable, we collapsed quarters 3 and 4 with the quarters 1 and 2 of the subsequent year as ‘season’, with the intent to model antigen shift and antigen drifts of the influenza virus. The 2011/12 seasonal influenza epidemics has started outstandingly late and was very mild [30]. Therefore, we ran models both with all seasons including season 2011/12, as well as with the recent seasons (2012/13 and later) excluding that season.

We performed statistical analyses and modeling with R in its version 4.0.4 (www.r-project.org). To assess periodicity, we subjected time series data to spectral analysis, using a maximum entropy method (MEM) in Python (https://github.com/martini-alessandro/Maximum-Entropy-Spectrum). The MEM is very well suited for short time series [31], however, an appropriate order of the underlying autoregressive process must be chosen in advance [32]. In our case, we decided for an order of 10 for a time series of 42 data points. This is in accordance with S Wust and M Bittner [33].

The function ‘bam’ from the r-package ‘mgcv’ was used to fit generalized additive models. Using the additive model fits, we estimated the number of influenza cases per 100,000 AOK clients per year, using the ‘predict’ function for all environmental stressors, with setting the other stressors to their medians, setting quarter = Q1, season to S14/15, and post code = D-68159. From these estimates, we computed the number of cases at the 5% and 95-percentiles of each respective stressor, keeping the values of the other independent variables constant, and we obtained the stressor-specific gain as the difference between predictions for these percentiles.

Given that the environmental stressor data are the result of a joint activity by many researchers, and that ICD-coded insurance data are highly standardized and controlled, there seems little potential for bias. To further reduce any bias, all data processing and statistical scripts, as well as interim results have been checked by at least two experienced programmers from the authors’ team.

## Results

**Fig. 2:**
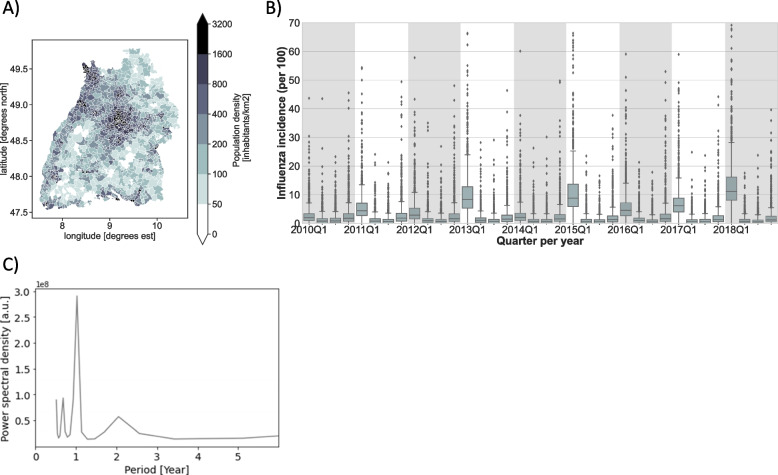
Geographical distribution of the cohort and influenza seasonality. **A** population density for each ZIP-code area in Baden- Württemberg. Population density data were derived from the DESTATIS’s Zensus2011 dataset. **B** boxplot reporting incidence per postcode for each quarter between of the years between 2010 and 2018. The y-axis has been cut at a value of 70, to allow better representation of boxes (**C**) Spectrogram of the data in (**B**). Note the pronounced peak at period = 1 year, and also the first harmonic at period = 2 years

The cohort of 3.85 million people resided within a total of 1194 postcodes that stretched out over at total of 35,712 km^2^. Population density was highly variable between postcodes (Fig. 2A), with a median of 249.5 inhabitants/km^2^, ranging from 2.4/km^2^ to 16,797/km^2^. Over the period of 9 years (36 quarters), a total of 513,404 influenza cases were observed, of which 53.6% occurred in quarter 1 (January to March), and 10.2%, 9.4% and 26.8% in quarters 2, 3 and 4, respectively. Overall, the influenza incidence amounted to 1482 cases per 100,000 AOK clients per year. A time series of influenza incidence is presented in Fig. 2B, and Fig. 2C depicts a spectrogram of these data. These plots reveal pronounced 1-year periodicity, in which incidences in quarter 1 consistently outnumber incidences in quarters 4, 2 and 3.

**Fig. 3:**
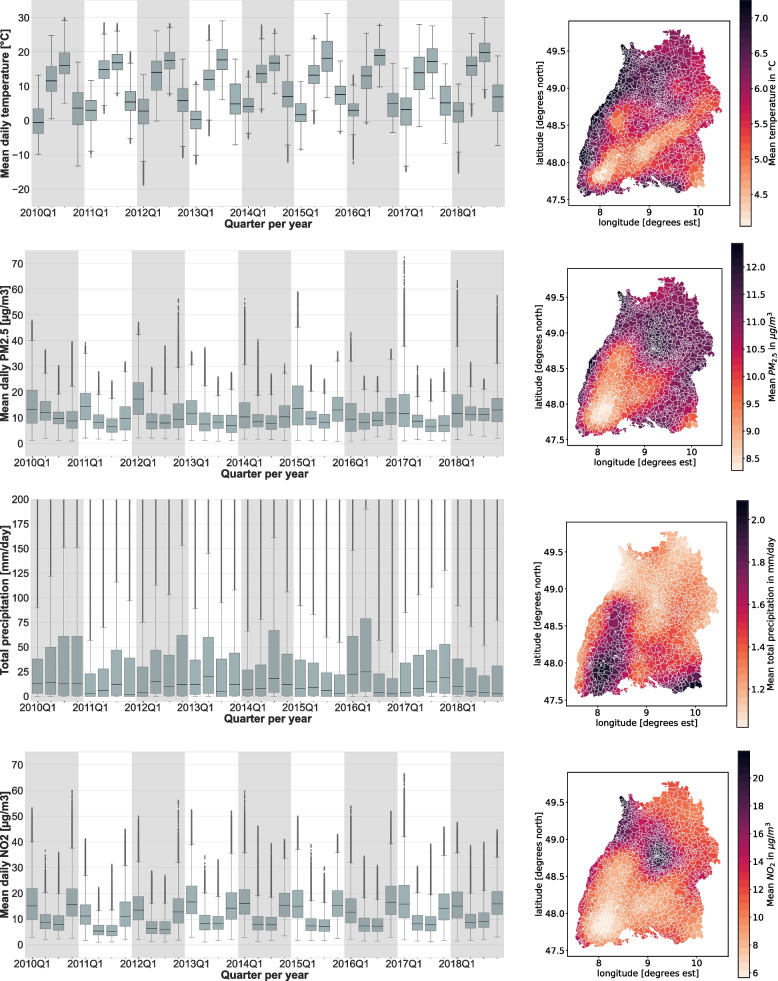
Seasonal and geographical variation in environmental stressors. On the left side, box plots for stressors per postcode and quarter reflect temporal variation from 2010 to 2018. For total precipitation, the y-axis has been cut off at 200 mm/day, so that box sizes have reasonable spread. To reflect spatial variation, the diagrams on the right side display the averaged stressor levels over the observation period 2010 to 2018, plotted in geographical postcode coordinates. The dataset used to produce the plots are those reported in the Table 1

Such quarterly variation with 1-year periodicity is also visible for the time series of Temperature, PM_2.5_ and NO_2_, but not for precipitation, as shown in the left panels of Fig. 3 and in Table3. The right panels of that figure depict spatial variation in the same stressors across the different postcodes in Baden-Württemberg.

**Table 3:** Means and percentiles of environmental data

	**PM**_**2.5**_	**NO**_**2**_	**Temperature**	**Precip-tation**	**O**_**3**_	**PM**_**10**_	**UV-Radiation**	**Vapor Pressure**
	[μg/m^−3^]	[μg/m^−3^]	[°C]	[mm/day]	[μg/m^−3^]	[μg/m^−3^]	[W]	[hPa]
**Mean Quarter 1**	14.08	15.99	2.01	2.89	42.55	18.71	9.45	5.92
**Mean Quarter 2**	9.96	8.53	13.29	3.59	69	13.56	23.77	11.16
**Mean Quarter 3**	8.98	8.37	17.59	3.51	63.95	12.64	21.84	14.52
**Mean Quarter 4**	10.85	15.78	5.77	3.57	30.55	14.59	6.43	8.16
**5-Percentile**	7.49	4.77	0.65	1.78	25.09	10.21	5.96	5.44
**Median**	10.28	11.61	9.05	3.23	52.7	14.27	15.5	9.78
**95-Percentile**	15.98	21.86	18.71	5.43	76.35	21.77	25.21	15.08

Quarterly mean values of environmental stressors (upper 4 rows), and percentiles and medians of the entire data set (lower 3 rows)

**Fig. 4:**
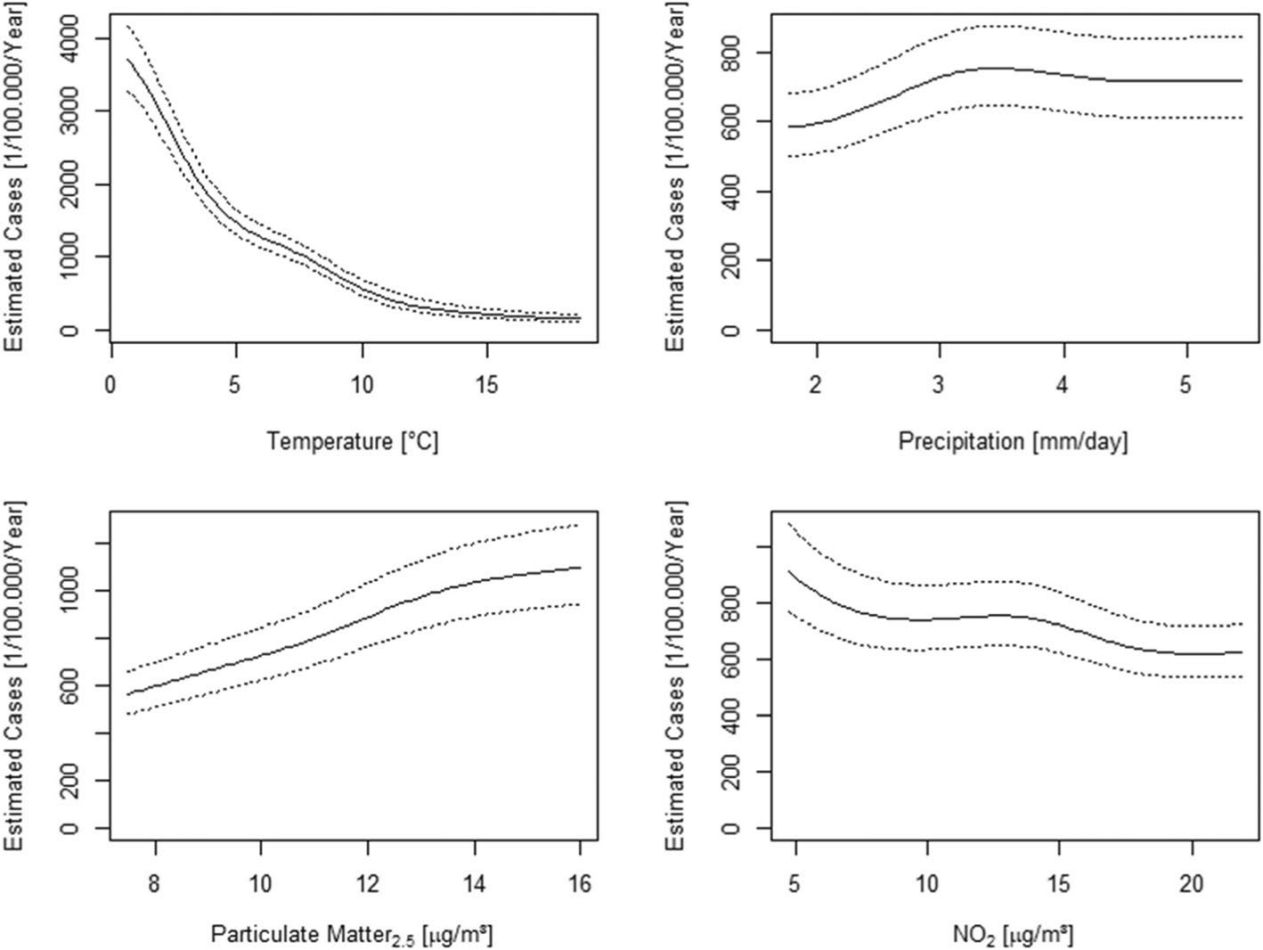
Estimated cases in response to variation of environmental stressors. Estimations (and 95% their confidence intervals) were obtained with the R-function predict for the 5- to 95-percentile intervals of Temperature, Precipitation, PM_2.5_ and NO_2_ from the restricted model for 2013–2018 data. Here we display curves for the restricted model (excluding quarter as independent variable) with recent season set (from season 2012/13 onwards). Note that the other 3 statistical models yielded trend curves that are very similar to those displayed here. Also, including interaction terms e.g. for temperature and PM_2.5_ had no obvious bearing on the trend curves

Statistical modeling yielded significant effects of temperature, precipitation, PM_2.5_, and NO_2_ for all four different models (all *P* < 10^–16^). When exploring the stressor-specific gains, effects were largest for temperature, ranging from 2639 (full model, all seasons) to 3499 (restricted model, recent seasons) cases per 100,000 AOK clients per year that can be attributed to temperature (Table 4). Plotting the estimated temperature effects revealed that the negative association between temperature and influenza incidence was most pronounced below 5 °C, and that the effect tapered off towards the higher temperatures (Fig. 4). The second largest effect was exerted by PM_2.5_, with a stressor-specific gain between 297 (restricted model, all seasons) and 502 (full model, recent seasons) cases per 100,000 AOK clients per year (Table 4). As can be seen from Fig. 4, the positive association was relatively steadily over the entire range of PM_2.5_ concentrations. Effects for precipitation and NO_2_ were in the order of 200 cases per 100,000 AOK clients per year only. Counterintuitively, however, NO_2_, was negatively related to influenza incidence (Fig. 4). It is also remarkable that the association for precipitation was only observed below 3mm/day.

**Table 4:** Attributable cases

	**Model**	**Restricted**	**Restricted**	**Full**	**Full**
	**Seasons**	**All**	**Recent**	**All**	**Recent**
Temperature	Prediction 5-Percentile	3113 (2814–3443)	3423 (3046–3846)	3315 (2994–3671)	3615 (3214–4067)
	Prediction 95-Percentile	474 (435—516)	396 (360—437)	187 (136—256)	116 (81—166)
	**Gain**	**-2639**	**-3027**	**-3128**	**-3499**
	**Relative Risk**	**0.152**	**0.116**	**0.0564**	**0.0320**
Precipitation	Prediction 5-Percentile	553 (500–612)	469 (415–529)	601 (520–696)	389 (325–467)
	Prediction 95-Percentile	760 (689—838)	638 (570—714)	743 (638—866)	476 (398—569)
	**Gain**	**207**	**169**	**142**	**87**
	**Relative Risk**	**1.37**	**1.36**	**1.23**	**1.22**
PM_2.5_	Prediction 5-Percentile	550 (497–608)	440 (390–497)	601 (516–699)	329 (271–398)
	Prediction 95-Percentile	984 (888—1091)	942 (829—1070)	897 (779—1034)	739 (617—886)
	**Gain**	**435**	**502**	**297**	**411**
	**Relative Risk**	**1.79**	**2.14**	**1.49**	**2.25**
NO_2_	Prediction 5-Percentile	950 (843–1069)	735 (638–847)	892 (761–1045)	535 (439–652)
	Prediction 95-Percentile	656 (600—717)	585 (529—647)	722 (628—829)	464 (397—542)
	**Gain**	**-293**	**-150**	**-170**	**-71**
	**Relative Risk**	**0.691**	**0.796**	**0.810**	**0.867**

Enlistment of estimated cases per 100,000 persons per year for variation in environmental stressors. For each of the four models, the estimated number of cases is given for the 5- and 95 percentiles of the environmental stressors: temperature; precipitation; PM2.5 and NO2, with the 95% confidence interval in brackets. For each stressor, the gain in estimated cases was computed as the difference between estimations for the 5- and 95-percentiles. Similarly, the relative risk is the 95%-to-5% ratio of case predictions. Full models include quarter as independent variable, and restricted models do not. Models with all seasons include all seasons from 2010/11 and later, whilst models with recent seasons include only the seasons 2012/13 and later

## Discussion

The important finding of our study combining Earth observation-based modeling with health insurance data is that seasonal variation in the incidence of influenza is attributable to temperature effects and to particulate matter. In this respect, our results are in line with previous *in-situ* urban studies [6, 7, 9, 34, 35] and extend the evidence to a mixed urban and rural cohort and to out-patients. However, associations with precipitation were at best moderate, and an incidence-promoting effect by NO_2_ cannot be supported on basis of our data. The associations found were robust across the four different statistical models used, which is particularly noteworthy with regards to the consistency between full and restricted models. The facts that the full models allowed quarter as an explanatory variable without direct involvement of the stressors, and that estimations did not deviate much when quarter was excluded as independent variable, suggest that the observed stressor effects reflect more than a mere temporal coincidence.

Even though our analyses found the strongest effect for environmental temperatures, the exact mechanisms by which temperature exerts the effects remain to be elucidated. Thus, as can be seen from Table 2, Pearson’s correlation coefficient between temperature and vapor pressure was 0.98, meaning that very similar results must be expected if temperature is replaced by vapor pressure. Indeed, previous work had suggested that vapor pressure rather than temperature per se is the effective agent that disrupts the influenza virus [14, 36]. It may also speak in favor of this idea that the observed relationship between influenza incidence and environmental temperature was strongly curvilinear (Fig. 4), which would be expected from Eq. 1 in this paper and Fig. 3 in J Shaman and M Kohn [14] if the temperature effect is mediated by vapor pressure. On the other hand, host susceptibility is known to be increased at low temperatures, e.g. via hampered interferon-β expression in respiratory cells and thereby blunted immune response [37]. And, as a third of many other alternative explanations, the temperature effect could also be partly mediated by UV radiation [38], with the latter being closely correlated to temperature (Table 2), and UV being well-known to inactivate viruses.

The results for precipitation suggest a comparatively moderate positive association that is levelling off above 3 mm/day (Figure), which is somewhat unexpected. Of note, as much as precipitation is related to relative humidity and vapor pressure, this observation is opposed to the purported anti-viral effects for the latter two [36]. The expected association has indeed been found by a recent study from Gwangju in South Korea [34]. Of note, precipitation in Gwangju is accentuated in the summer months, but relatively independent of quarters in Baden-Württemberg (Fig. 3, Table). Moreover, summers are approximately 10 °C hotter in Gwangju than in Baden-Württemberg. It therefore seems possible that precipitation may elicit anti-viral effects in humid-hot climates, but promote virus infections e.g. via compromising host defense mechanism in cold-humid climates.

The second-largest association with influenza incidence in our data was observed for PM_2.5_. Of note, collinearity of PM_2.5_ with other stressors in Table 2 was substantially smaller than for temperature (with the exception of PM_10_). It therefore seems that the observed association points towards a genuine PM_2.5_ effect. Literature identifies two different, but not exclusive, types of explanations, namely by stabilization of airborne virus particles *e.g.* against UV light, and / or by compromising the host’s defense mechanisms. Thus, it is well established that PM_2.5_ elicits inflammatory responses [39], which are apt to chronically compromise airways and foster infections as typically seen in asthma and chronic obstructive pulmonary disease.

The pertinent question is how out-door PM_2.5_ levels, as used here, could affect people’s health when people spend most of their time indoors. Thus, an analysis of 2010 found that only 1.04 h per day are outdoor during weekdays, and only 1.64 h per day during weekends [40]. Indoor PM results from infiltration from outdoors, and from primary and secondary indoor sources [41, 42]. Primary sources include all types of combustion (*e.g.* for heating and cooking, but also including smoking), but also sources such as laser printing devices, handicrafts and organic aerosols from human and animal sources. Secondary indoor sources include particles infiltrated from outside that chemically react with particles from indoor sources. B Wang, Y Liu, Z Li and Z Li [43] have demonstrated that such indoor sources, in particular coal combustion have a strong bearing on influenza infections. In addition, it is well established that indoor PM significantly depends on outdoor PM [41]. However, virtually all buildings in Baden-Württemberg afford central heating, and heating affects indoor PM levels in two ways. Firstly, by directly fostering the convection-driven resuspension of PM, and second by reducing relative humidity and thereby hampering PM suspension [44]. Therefore, although speculative at this moment, there is a possibility that outdoor temperature impacts on influenza via heating-related seasonal variation in indoor PM levels.

Finally, the present study does not support the view of NO_2_ being a major driver for influenza, an idea that was put forward with regards to the current SARS-CoV2 pandemic [45, 46]. Instead, we observed a very moderate negative, rather than a positive, association between NO_2_ levels and incidence (Fig. 4), which may more likely be due to statistical collinearity with temperature or PM2.5 than the hallmark of NO_2_-related salutogenesis. Naturally, differences could exist with regards to virus-environment interactions between influenza and SARS-CoV2. The mechanism that have to date been proposed for mediating environmental stressor effects are very similar for these two viruses. Thus, observations made for influenza infections are certainly of interest also for a better understanding of the Covid-19 pandemic. Many studies world-wide have reported modulation by environmental stressors, albeit with mixed results [47–50]. Thus, even though environmental factors certainly did not fully determine infection rates in the early days of the pandemic [51], it does seem reasonable to consider environmental factors for disease control. This applies foremost to factors that can be modified with justifiable effort.

An important limitation of the present study is the limited spatial resolution (postcode area), and also the lack of information regarding the sojourn of individuals. Whilst it has become technically possible to monitor environmental exposure on a personal basis [52], the cost involved and the need of consenting participants would preclude sample sizes as in the present study. Therefore, given the lack of personal mobility data, the coarse graining of spatial information is not necessarily a disadvantage, in particular because spatial variation of stressor levels was fairly high in our data set (Fig. 3). The environmental data used for this research work have a native horizontal resolution of 0.1° × 0.1°, that, at the latitudes of interest corresponds to an area of approximately 55 km^2^. The main advantage of using these datasets consists in having a constant and reliable data availability for the spatial and temporal domain considered. Furthermore, the datasets used are all defined as reanalysis. This implies that a consistent quality check by comparison with observational data and their assimilation in the datasets has already been carried out. However, the cohort design of this study required a spatial aggregation of the environmental to the ZIP-code areas in Baden-Württemberg. Many of these areas are smaller with respect to the resolution of the dataset.

Another weakness of the present study is the unknown proportion of diagnoses that are based on clinical inspection only, with no confirmation by laboratory testing. However, obtaining confirmatory testing would have been impossible in the current study setting, and it would be extremely resource-demanding in any study sample of that magnitude with 34.7 million observed person-years and more than half a million diagnosed cases. Moreover, it has been demonstrated that clinical surveillance for influenza is synchronous with laboratory surveillance [5], suggesting that clinical diagnosis is a valid proxy of influenza infections.

Finally, the question of generalizability of the results arises. Whilst AOK used to be the designated health insurance for the working class until 1996, nowadays there is a free choice of health insurance in Germany. We regard the predominant inclusion of a working-class cohort as a strength of our approach, because it reduces influences from socio-economic status, and because populations of lower socio-economic status are typically more affected by environmental health issues. Therefore, a slight bias exists in the present cohort with relatively higher fractions of working class in the older as opposed to the younger age groups. However, the morbidity-related risk structure compensation, which is a monetary mechanism to enforce equity between German insurance companies, is very similar between AOK-BW and the average of the other German health insurance companies, which gives us confidence that the cohort underlying this study represents Germany’s population to a large degree.

## Conclusions

Our study is in line with and extends previous studies with *in-situ* ground measurements reporting strong associations between the environmental stressors temperature and particulate matter upon influenza incidence. In particular, our study suggests that such associations exist also for a region with temperate climate encompassing urban as well as rural areas. However, the present results put previous reports on associations with precipitation and NO_2_ into perspective. Whilst the true effects of temperature observed here are, at least partly, exerted via collinear effects such as ultraviolet radiation, ozone, and other routes. In contrast, effects observed for particulate matter were statistically less collinear with other stressors. We, therefore, suggest that reduction of particulate matter could offer leverage for public health policies in relation to influenza and other viral infections. Future studies should aim at gaining information on indoor levels of particulate matter, and at gaining more individualized information on exposure levels. In addition, it should be analyzed which costs can be directly linked to the environmental stressors. The DLR and AOK BW want to continue their partnership for further analyses.

Finally, this investigation also demonstrates that Earth observation-based modeling in combination with health insurance data constitutes a powerful concept for public health studies. In the medium and long term, there is the vision for a location-independent and individualized risk prediction that will decisively advance healthcare and prevention and draw the population of Baden-Württemberg to the effects of climate change adequately prepared and protected.

## Data Availability

The data that support the findings of this study are available on request from the corresponding author JR. The data are not publicly available due to the high standards of data protection for health data.

## Abbreviations

ADS: Athmosphere data store (of Copernicus)
AOK: Allgemeine Ortskrankenkasse (public health insurance company)
CAMS: Copernicus atmosphere monitoring service
CDS: Climate data store
C3S: Copernicus climate change service
DLR: German Aerospace Center
EEA: European environmental agency
MEM: Maximum entropy method
NO_2_: Nitrogen dioxide
O_3_: Ozone
PM: Particulate matter
PM_10_: Coarse particular matter
PM_2.5_: Fine particular matter
SO_2_: Sulfur dioxide
UV: Ultraviolet
